# Supplementary material for: Downregulation of Ribosomal Protein Genes Is Revealed in a Model of Rat Hippocampal Neuronal Culture Activation with GABA(A)R/GlyRa2 Antagonist Picrotoxin
Source: Cells. 2024 Feb 23;13(5):383. doi: 10.3390/cells13050383 (PMC10930765; doi:10.3390/cells13050383)
Supplement: Supplementary file 1 [file cells-13-00383-s001.zip › Supplementary Document S1.pdf]

Command examples with parameters, used in the study:

Guppy basecalling

guppy_basecaller -i in -s out -c dna_r9.4.1_450bps_hac.cfg -x "cuda:0" --compress_fastq

NanoFilt:

NanoFilt -q 10 -l 200 --headcrop 10 --maxlength 10000 in.fq > out.fq

Minimap2/samtools:

minimap2 -t 8 -N 100 -p 1 -ax map-ont transcriptome.fa in.fq | samtools view -bh > out.bam

minimap2 -ax splice -uf --cs --MD -t 6 --junc-bed jnc.bed genome.fa in.fq > out.sam

samtools sort -o out_sort.sam -O bam -@ 6 out.sam

Salmon quant:

salmon quant --libType A -a in.bam -t transcriptome.fa --noLengthCorrection --noErrorModel -o out -p 6

FeatureCounts:

featureCounts -T 4 -O --fraction -a gtf.gtf -L --primary -o out.txt in1.bam..in.bam

Flair assembly:

python bam2Bed12.py -i in.bam > in.bed

python flair.py correct -g genome.fa -f gtf.gtf -q in.bed -o corrected.bed

cat corrected_1.bed.. corrected_20.bed > corrected.bed

flair.py collapse -f gtf.gtf -g genome.fa -q corrected.bed -t 6 -o relaxed -r 1.fq..20.fq

FLAMES assembly:

```
FLAMES/python/bulk_long_pipeline.py \
   --gff3 gtf.gtf \
   --genomefa genome.fa \
   --outdir out_FLAMES \
   --fq_dir FLM
```

Stringtie assembly:

stringtie -L -p 4 -G gtf.gtf -o out_1.gtf in_1.bam

stringtie --merge -G gtf.gtf -o GTF_merged.gtf out_1.gtf..out_20.gtf

Gffcompare count locus-level statistics:

gffcompare –r subset.gtf genome.fa raw_assembly.gtf

Gffcompare merge:

gffcompare -D -S --strict-match -C -s genome.fa rn7.gtf raw_assembly.gtf

DFAM search:

nhmmer -o Tab_out --tblout Hits_out --aliscoresout Scores_out --incE 0.0000000001 --dna --cpu 8 Dfam_curatedonly.hmm.gz transcripts.fa
