# Supplementary material for: Downregulation of Ribosomal Protein Genes Is Revealed in a Model of Rat Hippocampal Neuronal Culture Activation with GABA(A)R/GlyRa2 Antagonist Picrotoxin
Source: Cells. 2024 Feb 23;13(5):383. doi: 10.3390/cells13050383 (PMC10930765; doi:10.3390/cells13050383)
Supplement: Supplementary file 1 [file cells-13-00383-s001.zip › Supplementary figures.pdf]

cells

Supplementary Figures

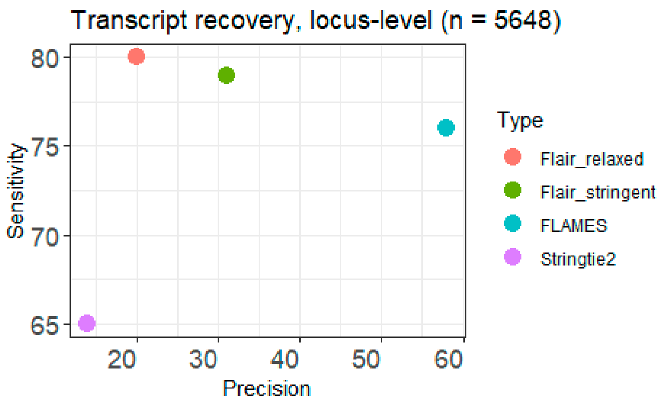

Figure S1. Comparison of different long-read transcriptome assembly pipelines by their ability of restoring sequence of 5648 most abundantly expressed transcripts using the Rn6 genome assembly.

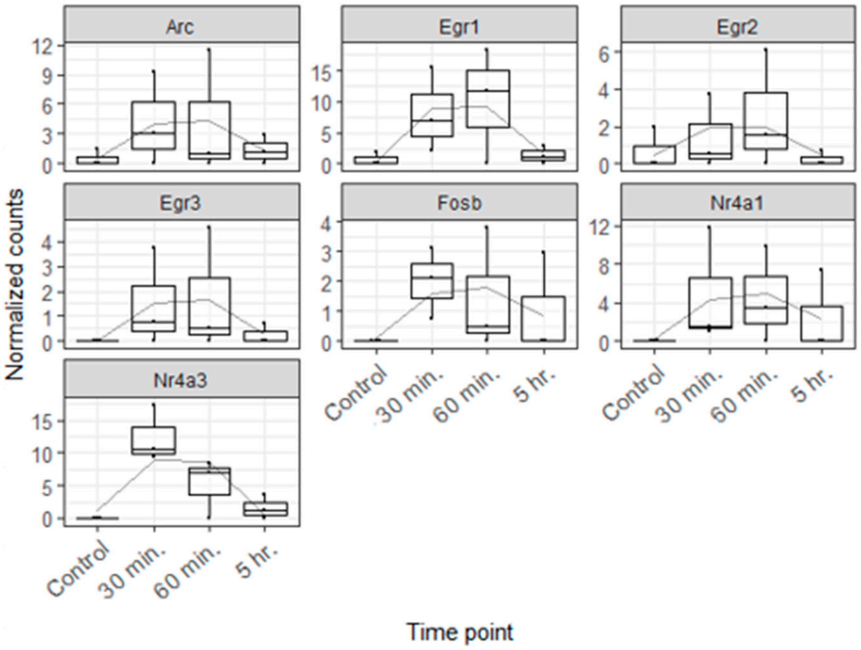

Figure S2. Expression dynamics of known IEGs which did not pass the significance threshold for any group (AM±SD).

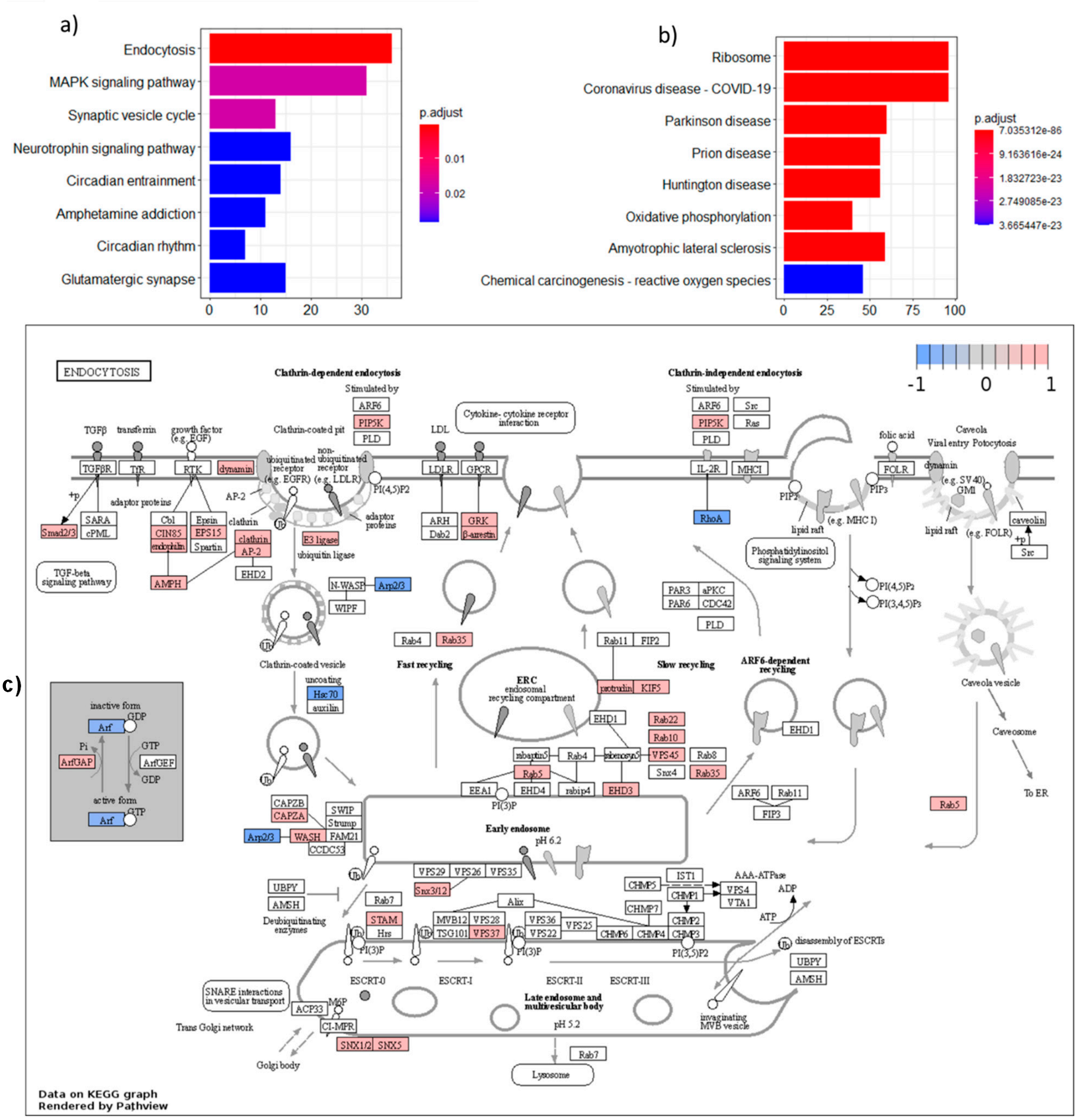

Figure S3. KEGG pathways for a) up-regulated and b) down-regulated genes at 30 min after the PTX application. c) Visualisation of the endocytosis pathway. Genes downregulated in the 30 min dataset are shown in blue, upregulated ones in red.

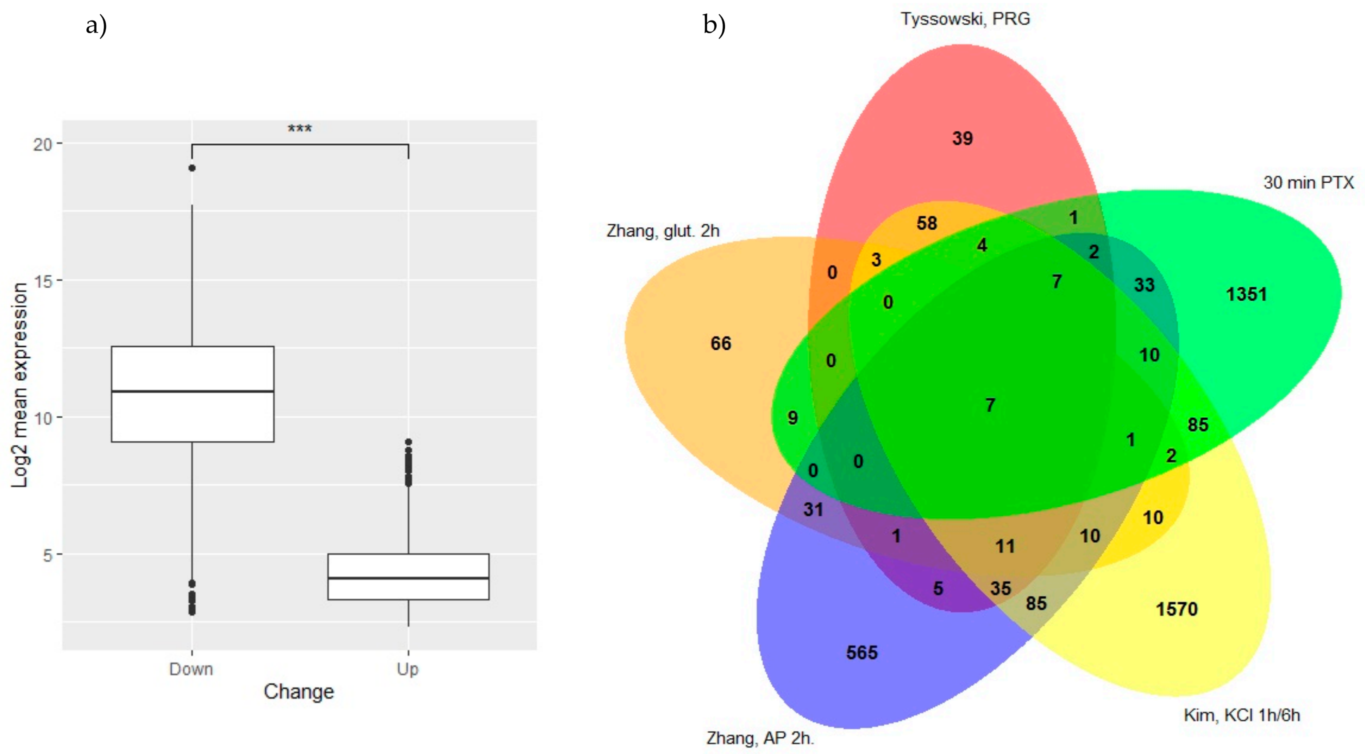

Figure S4. a) Baseline expression levels (median-of-ratios normalized counts) averaged among all genes in up-regulated and down-regulated gene groups ("up-regulated" and "down-regulated" here mean expression level changes registered in samples with 30 min PTX treatment compared with control). AM±SD, *** - p < 0.001, Mann-Whithey U-test. b) Venn diagram of all genes, expression of which was reported to significantly change after different culture stimulation protocols in [71,76,78] and in the present work (30 minutes PTX, green ellipse). PRG - primary response genes, glut. – glutamate, AP – action potential bursting by bicuculline.

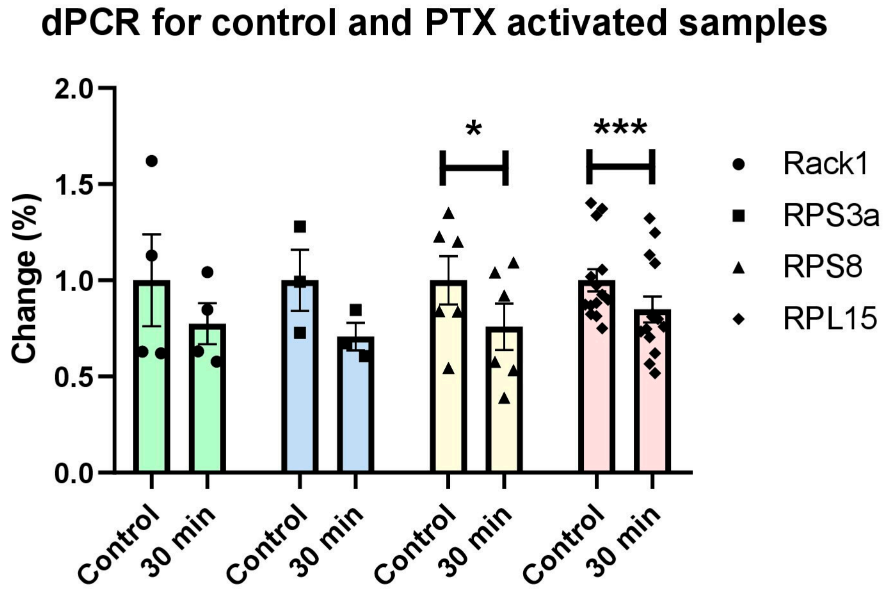

Figure S5. dPCR results for selected genes encoding ribosome and ribosome-associated proteins. Control group: *n*=4 for *Rack1*; *n*=3 for Rps3a; *n*=6 for *Rps8*; *n*=15 for *Rpl15*; "30 min" PTX activation group: n=4 for *Rack1*; n=3 for Rps3a; n=6 for *Rps8*; n=15 for *Rpl15* biological replicates. Expression levels of genes of interest were normalized to the average *Hprt* expression in the same sample. The results are present as proportional change in gene expression ± SEM normalized to the control group value; * - $p < 0.05$, *** - $p < 0.001$, Wilcoxon signed rank test.

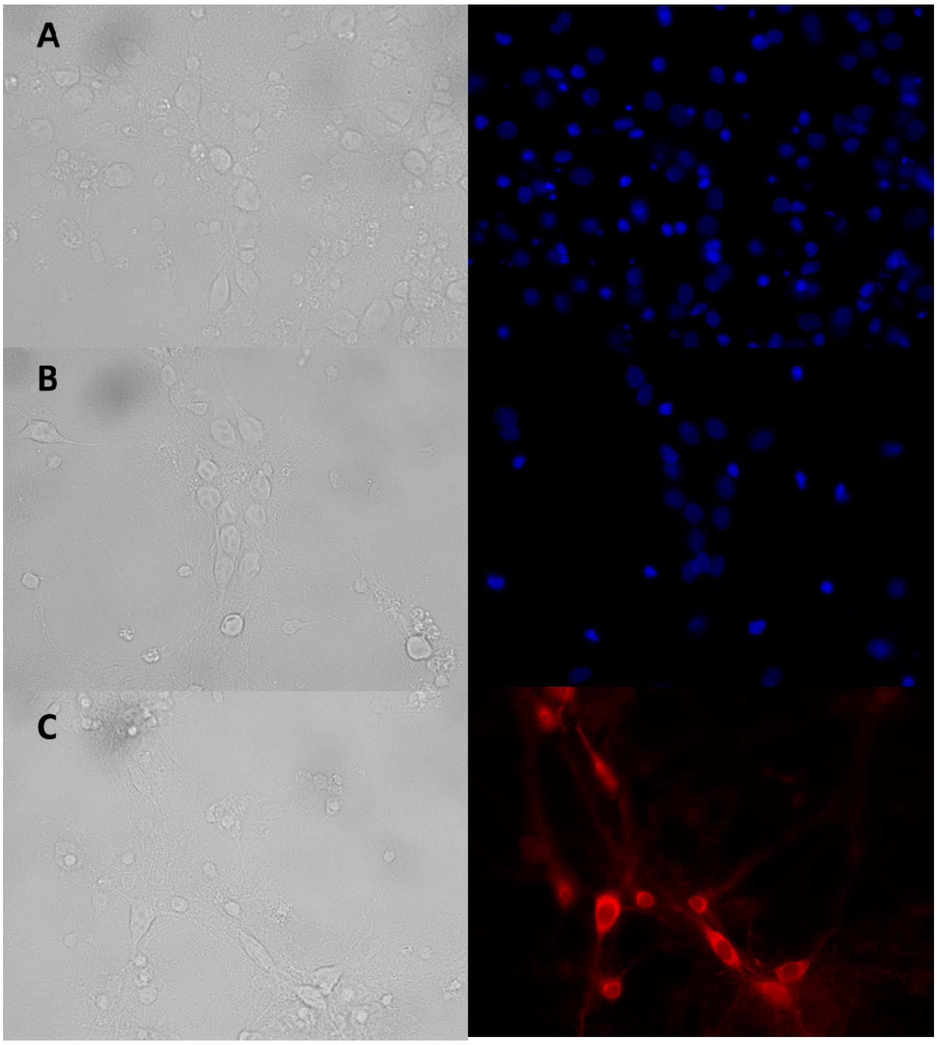

Figure S6. Primary cultures of rat hippocampal neurons at DIV15. a) control; b) incubation with PTX for 30 min. Absence of morphological changes in neurons after exposure to picrotoxin; Left - bright field microscopy; right - nuclei stained with DAPI (blue); c) control culture stained with antibodies to alpha subunit of CaM kinase II (CaMKII-alpha; red). Keyence BZ-9000E; oil immersion x60 microscope objective.
